# Hybrid Graphene-Metal Oxide Solution Processed Electron Transport Layers for Large Area High-Performance Organic Photovoltaics

**DOI:** 10.1002/adma.201304780

**Published:** 2014-01-02

**Authors:** Michail J Beliatis, Keyur K Gandhi, Lynn J Rozanski, Rhys Rhodes, Liam McCafferty, Mohammad R Alenezi, Abdullah S Alshammari, Christopher A Mills, K D G Imalka Jayawardena, Simon J Henley, S Ravi P Silva

**Affiliations:** Advanced Technology Institute, University of SurreyGuildford, GU2 7XH, UK

**Keywords:** core/shell nanoparticles, graphene hybrid materials, metal oxides, organic photovoltaics, charge transport, interface engineering

Organic photovoltaics (OPVs) are receiving considerable attention due to their potential as a source of renewable energy.^1^,^2^ Compared to conventional silicon-based photovoltaics, they are lightweight, flexible, and are compatible with low-temperature solution-based processing techniques resulting in a considerable cost reduction over contemporary devices. In the last few years, high performance polymer solar cells based on bulk heterojunction (BHJ) structures have been reported with efficiencies up to 10% providing impetus for the next generation organic photovoltaic devices leading to 4^th^ generation (4G) hybrid solar cells^3^ and their successful commercialization.^3^–^7^

Despite these recent achievements in device efficiency, the mass production of OPVs require that solution–processed, low-cost materials can be obtained in sufficient quantities to support large-scale manufacture. Organic solar cells are stratified structures including a photoactive layer, interfacial buffer layers, and electrodes. While a substantial effort is underway in the scientific community to improve the absorption spectrum and charge generation in the photoactive layer,^8^,^9^ the fabrication of efficient interfacial layers^10^,^11^ used between the active layer and the electrodes requires equal scientific endeavor. The photogeneration of excitons and their dissociation is mainly dominated by the optical and electrical properties of the active layer whilst the charge transport-extraction process depends critically on the interfacial carrier transporting layer (CTL) between the active layer and the metal electrodes.^12^ Adequate CTLs can prevent recombination of electron and holes by providing selective and fast carrier transportation.^13^ Thus, they have a direct effect on the overall performance of OPVs. Recently, engineering novel CTLs have received significant attention by researchers seeking to improve device stability to air and moisture exposure, solution process-ability at low temperatures, and to achieve desirable band structures in order to improve performance of the OPVs.^12^–^14^ At present, the state-of-the-art OPV devices use solution processed poly(3,4-ethylenedioxythiophene):poly(styrenesulfonate) (PEDOT:PSS) with plasmonic nanoparticles^10^,^15^–^17^ as a hole transport layer (HTL) and thermally evaporated calcium,^18^ bathocuproine (BCP) or metal oxides (MO) from precursors such as TiO_x_^19^ and ZnO_x_^20^ as the electron transport layer (ETL). Furthermore, ETLs containing MOs can function as optical spacers^21^,^22^ which serves to enhance the distribution of light in the photoactive layer and further increase device performance. However, metal oxide layers synthesized via sol-gel,^14^,^20^ pyrolysis^23^ or hydrothermal^24^ methods require high annealing temperatures to promote crystallization which is incompatible with flexible substrates. An essential aspect towards fully solution processed OPVs on flexible substrates, is the design of bespoke high performance solution processed electron transport layers which exhibit high electrical mobility, good optical transparency and low fabrication temperatures.

In order to address these problems, we study the use of low cost commercial off-the-shelf, metal oxides (TiO_2_ and ZnO) with facile availability, compatibility with roll to roll deposition, well-defined crystal structure and high transparency. Furthermore, we synthesize novel hybrid ETL materials by chemically modifying these metal oxides with reduced graphene oxide (RGO) layers, thereby tailoring their electrical and optical properties to entail the optimal performance. We utilize these new materials to fabricate solar cell devices and study their performance as ETLs with the air stable polymer system: poly[N-9″-hepta-decanyl-2,7-carbazole-alt-5,5-(4′,7′-di-2-thienyl-2′,1′,3′-benzothiadiazole)] PCDTBT – [6,6]-phenyl C70-butyric acid methyl ester (PC_70_BM). Using these hybrid materials as ETLs, we achieved performance enhancement of 8.38% compared to solar cells utilizing pristine metal oxides as ETLs. Record high power conversion efficiency (PCE) of 6.72% and 6.57% on single devices with active area size (28.3 mm^2^) is demonstrated using the hybrid ZnO-RGO and TiO_2_-RGO as ETLs respectively. Module cells with large active area (3920 mm^2^) using ZnO-RGO as ETL operated at PCE of ∼3% demonstrating ∼20% improvement compared to the reference cell with pristine TiO_2_ for ETL is also reported. The output power from large solar cells was sufficient to power up a commercial LED mounted on a plastic substrate, providing evidence that this type of OPVs can be used to power small portable electronic devices, such as autonomous sensing systems.

Electron microscopy images of the RGO loaded TiO_2_ and ZnO before dilution in methanol are displayed in **Figure** **1**a,b and c, d respectively. Clear evidence of the metal oxide-graphene particles in a core-shell configuration can be observed from the SEM (Figure 1a,c) and TEM images (Figure 1b,d). Both metal oxides are covered with a uniform ∼3 nm shell indicating that approximately nine layers of RGO were attached to the surface of the MO particles. Raman studies were performed on both the pristine and graphenated versions for all metal oxides (Figure1e,f) to verify the nature of the hybrid materials. The peaks at 438, 598 and 797 cm^−1^ of pristine TiO_2_ are the B_1g_, A_1g_, and E_g_ phonon vibrations in the lattice, characteristic for TiO_2_.^29^–^32^ Similarly, for pristine ZnO the characteristic phonon peaks appear at 450 cm^−1^ (E_2_^high^), 571 cm^−1^ (LO), 667 cm^−1^ (P (plasma)), 894 cm^−1^ (2E_2_^high^), 1035 cm^−1^ (2LO), 1800 cm^−1^ (3LO), 2411 cm^−1^ (4LO) and 2900 cm^−1^ (5LO).^33^–^37^ Distinctive graphene signature D and G bands were observed at 1322 cm^−1^ and 1599 cm^−1^ respectively in Raman spectra of graphenated TiO_2_ and ZnO metal oxide films confirming the materials’ composition.^38^ The G peak arises from the phonon vibration with E_2g_ symmetry and denotes the sp^2^ bond configuration,^39^ while the appearance of a strong D peak is associated with the breathing mode of k-point phonons of A_1g_ symmetry resulting from large disorder on the sp^2^ hybridized hexagonal configuration of carbon atoms in the sheet due to increased number of defects.^38^,^40^ This could be attributed to excess damage of RGO flakes caused at the harsh environment in the autoclave during the vigorous process of reduction hybridization and to irreversible defects induced during the oxidation process of initial graphite powder. For both metal oxides, their characteristic Raman peaks are decreased substantially in the MO-RGO versions, a possible explanation could be due to energy absorption in the RGO flakes thus lowering phonon vibrations excited within the metal oxides.

**Figure 1:**
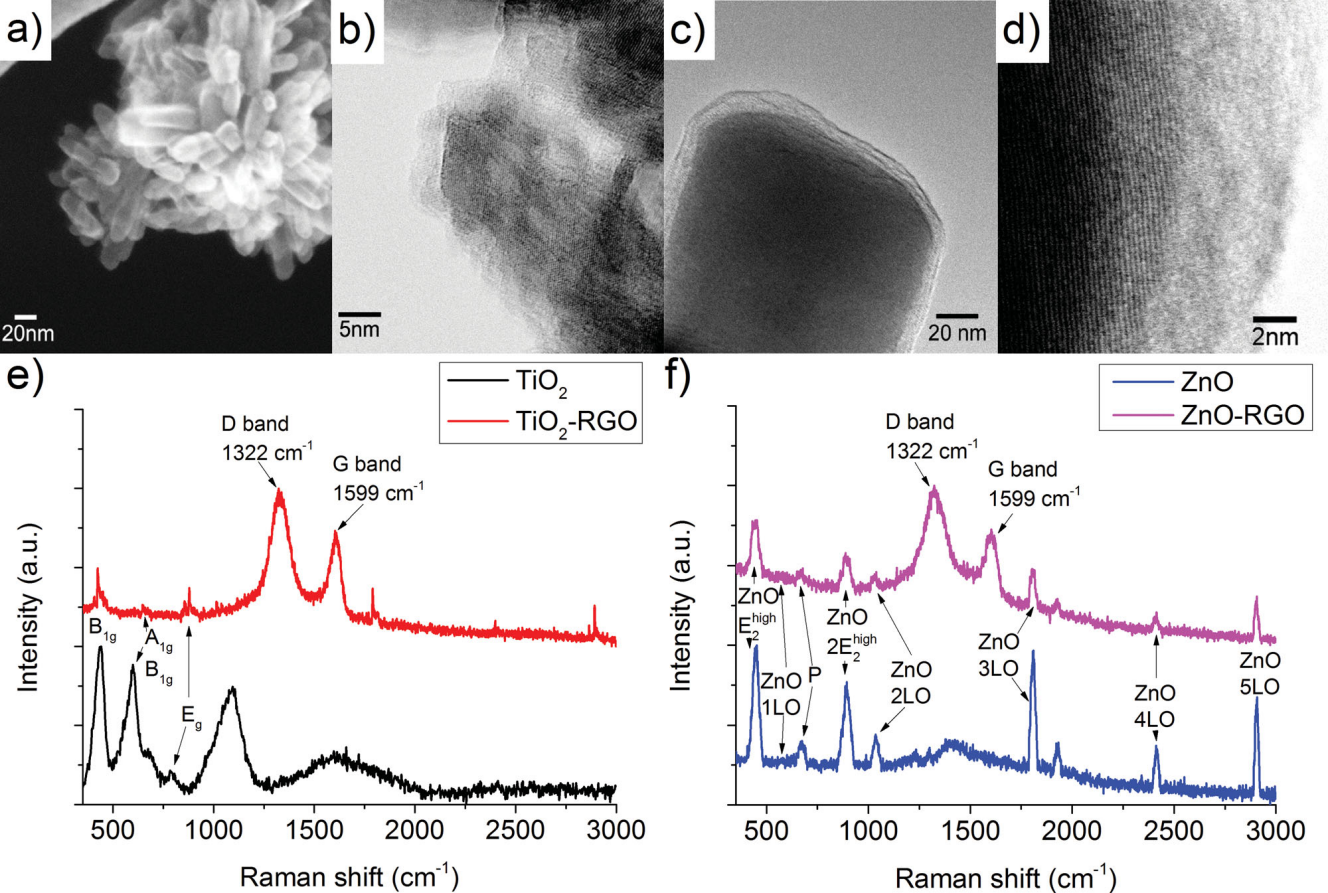
a,b) STEM images of TiO_2_-RGO, c-d) STEM of ZnO-RGO, e) Raman spectra of TiO_2_ (P25) [lower line] and TiO_2_-RGO with [upper line], f) Raman spectra of ZnO [lower line] and ZnO-RGO [upper line].

FTIR measurements on pristine and RGO loaded TiO_2_ show successful hybridization between the MO and RGO (see Supporting Information). By comparison, the sample of TiO_2_ loaded with RGO exhibits stronger, broader peaks with slightly shifted wave numbers. In particular, the Ti-O-Ti peak has increased in breadth (to ∼1000 cm^−1^) and intensity; this can be attributed to the combination of Ti-O-Ti and Ti-O-C stretching (which is centered at ∼798 cm^−1^)^41^ suggesting that the TiO_2_ has successfully bonded to the reduced graphene oxide sheets. Previous investigations have determined that TiO_2_ powders can undergo charge transfer reactions between hydroxyl groups on the TiO_2_ and carboxylic acid groups,^42^ which are known to be present on the surface of graphene oxide,^43^ indicating that during hydrothermal reduction the GO has bonded covalently to the TiO_2_ nanostructures. Furthermore, we show the expected reduction to almost zero of the C-O bond in the XPS spectrum centered at 286.9 eV in GO when reduced (RGO) (see Supporting Information), showing a high speed route to the bond hybridization between the RGO and MO.

OPV devices with ETLs from pristine metal oxides and hybrid RGO materials were fabricated to evaluate their effect on device performance. The current density versus voltage (*J–V*) and the external quantum efficiencies (EQE) in addition with the device structure and the materials’ energy band diagram for the OPVs are displayed in **Figure** **2**. All devices were fabricated using a non-inverted structure. The best TiO_2_-RGO device gave a V_oc_ of 0.9 V and a J_sc_ of 11.66 mA cm^−2^, with a FF of 62.07% and a PCE of 6.57%. The improvements seen for the MO-RGO devices hold true across multiple devices (see Supporting Information). The best device with ZnO-RGO show significant enhancement in J_sc_ reaching 12.54 mA cm^−2^ and an improved V_oc_ of 0.91 V leading to a record PCE of 6.72%. This indicates that the energy levels aligned successfully promoting an efficient electron extraction from the device. This hypothesis is supported by the conductivity values acquired from electron only devices for TiO_2_ (43.61 mS cm^−1^), TiO_2_-RGO (46.67 mS cm^−1^), ZnO (47.92 mS cm^−1^) and ZnO-RGO (56.72 mS cm^−1^) (see Supporting Information). The device electrical characteristics with graphene loaded ETLs are observed to be improved by a factor of 1.18 and 1.08 for ZnO and TiO_2_ respectively compared to devices with pristine MO's (Table 1). This indicates that the high PCE for the ZnO-RGO based device is directly influenced by the efficiency of charge extraction – collection mechanism.

**Table 1:** OPV peak electrical characteristics for each single cell device with the different ETL materials

ETL	V_oc_ (V)	J_sc_ (mA cm^-2^)	FF (%)	PCE (%)	R_s_ (Ω cm^2^ )	ETL Conductivity (mS cm^-1^)
TiO_2_	0.90	11.64	60.39	6.39	35.74	43.61
TiO_2_-RGO	0.90	11.66	62.07	6.57	25.33	46.67
ZnO	0.89	11.03	61.20	6.20	58.19	47.92
ZnO-RGO	0.91	12.54	58.91	6.72	24.43	56.72

**Figure 2:**
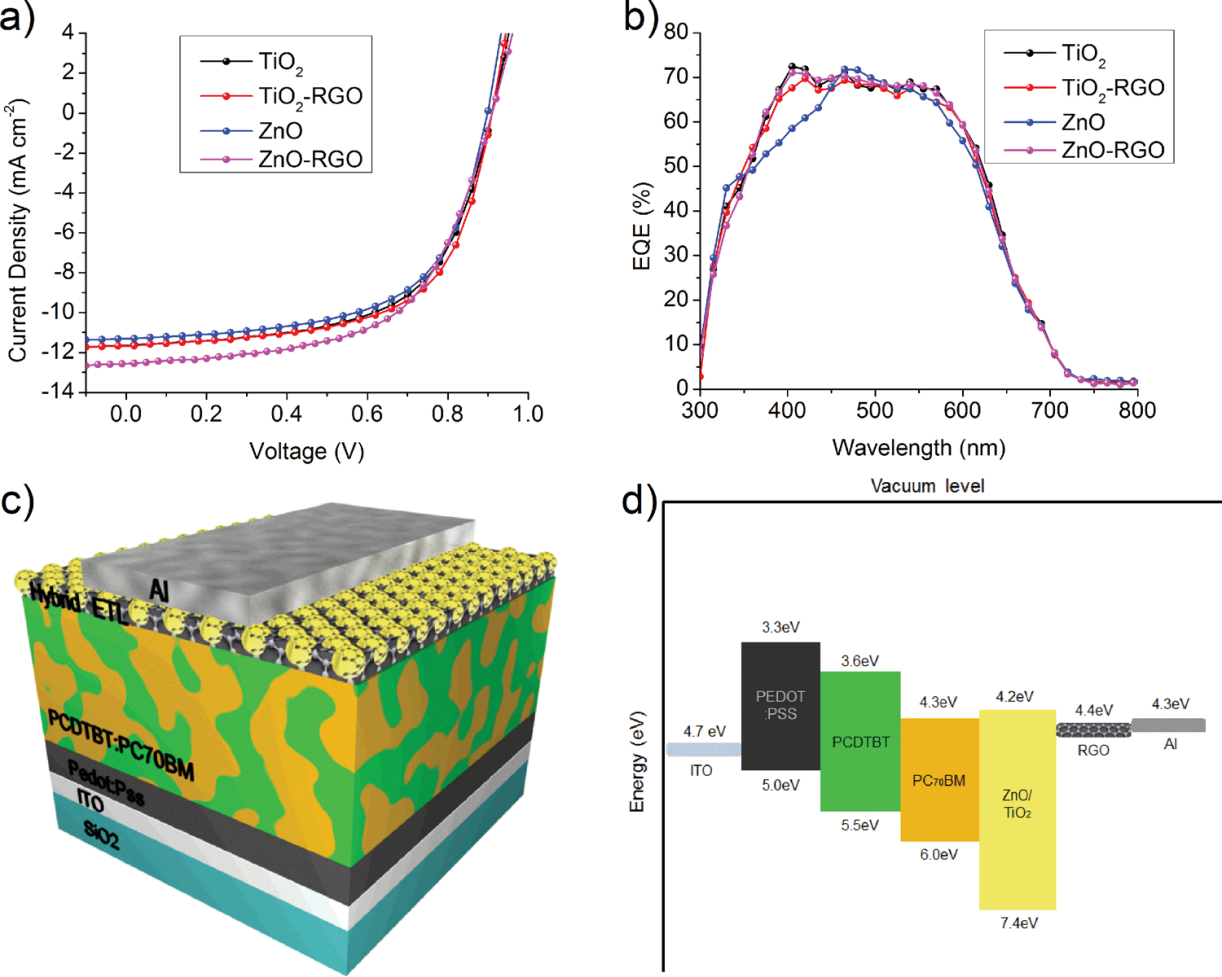
a) *J–V* curves of the OPV devices with four different materials (TiO_2_, TiO_2_-RGO, ZnO, ZnO-RGO) for ETL, b) EQE, c) the device structure, d) band diagram for the materials used in OPV devices.

The wide band gap of ∼3.2 eV and the valence band of ∼7.4 eV for both TiO_2_ and ZnO contributes to form an efficient hole blocking layer while the relatively well matched energy levels of the TiO_2_ and ZnO conduction bands match well with the work function of graphene (∼4.4 eV), forming an efficient energy transfer layer for fast electron extraction.

The module cell was fabricated using ZnO-RGO as ETL resulting to a PCE of ∼3% (see Supporting Information).

To obtain additional insight into the device performance, we examined the morphology of the metal oxide derived films. Atomic Force Microscopy (AFM) was used to examine the MO films on top of the active layer, before the back electrode deposition, in order to obtain the topographies shown in **Figure** **3**. Uniform films with all ETL materials composed of MO nanoparticles are observed. For pristine MO films cast from as-diluted solutions (as described in materials synthesis), the root mean square (RMS) roughness and maximum height were 0.7 nm, 4.2 nm for TiO_2_, while for ZnO these values were 0.59 nm and 3.38 nm. For the RGO-loaded MO films the equivalent values are 0.82 nm, 4.93 nm for RGO-TiO_2_ and 0.63 nm, 3.74 nm for RGO-ZnO. Although the films made with the commercial MO nanoparticle powders are similar in roughness to those films derived from sol-gel based precursors in other studies,^20^ intriguingly the commercial MO powders used here exhibit enhanced performance. This is ascribed to better crystallinity of the MO nanoparticle powder compared to the precursor, which contributes to improved charge extraction. Films made with TiO_2_ composites show a higher roughness compared to those of ZnO, as shown in Figure 3, a known factor which affects the performance of devices. It can be seen that the ETLs made with the hybrid graphene-MO materials are rougher to that ETLs using pristine MO. However, loading of RGO improves the conductivity of the film, evidenced by the decreased series resistance (R_s_) observed in the single cell devices (Table1). This decrease in R_s_ leads to ∼8% improvement in the efficiency of devices with hybrid ETLs. The absorption spectra of different ETLs within devices of the same architecture are shown in Figure 3e. The absorption spectra are very similar for all devices indicating there is minimum absorption from the ETLs; this suggests that it does not interfere with the ETL's secondary function as an optical spacer.^4^,^21^ As a result, the enhanced efficiencies observed with the RGO-MO hybrid ETLs can be ascribed primarily to their improved electrical characteristics.

**Figure 3:**
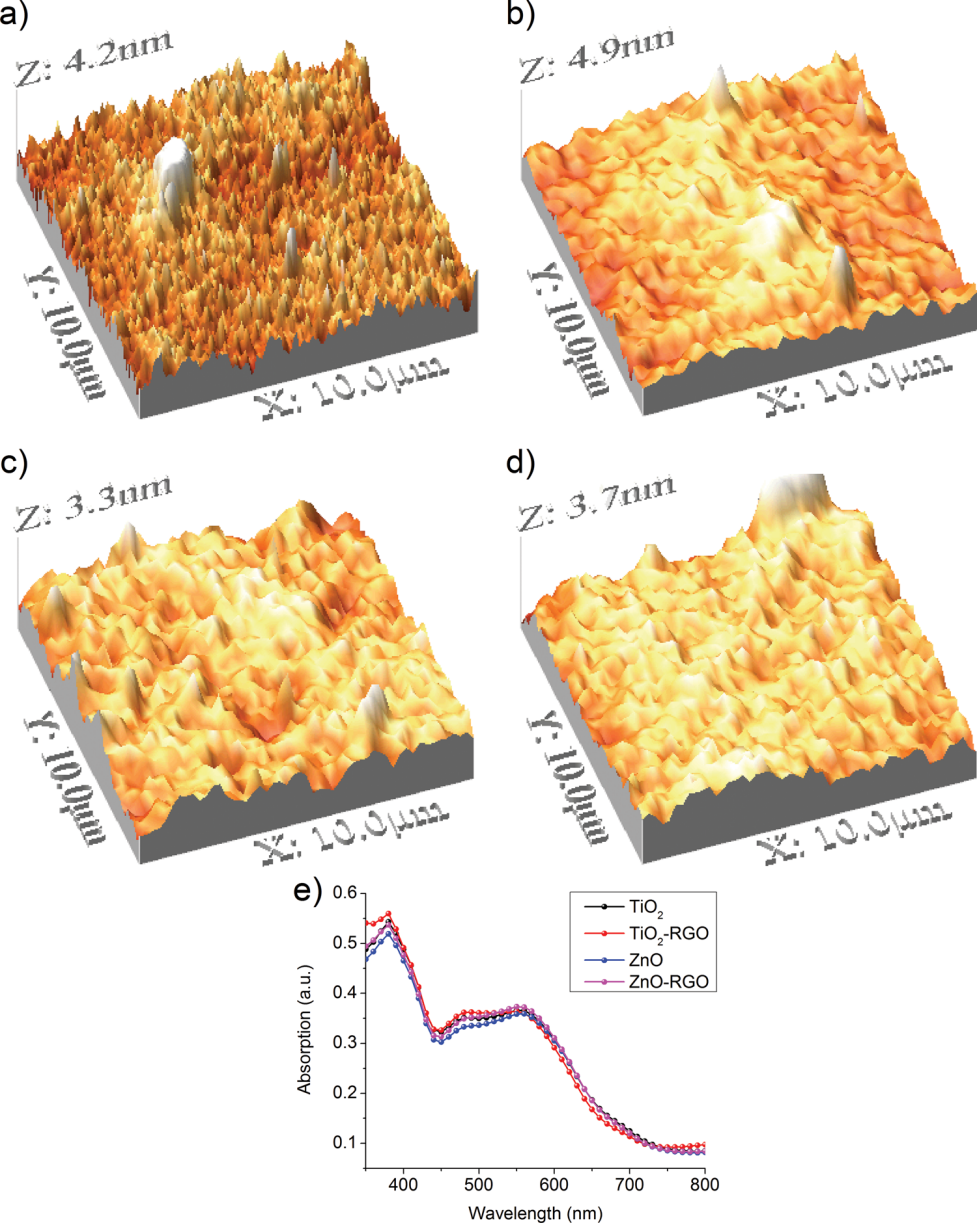
AFM micrographs of the morphologies of different ETL materials on top of the photoactive layer a) TiO_2_, b) TiO_2_ – RGO, c) ZnO, d) ZnO-RGO and e) the absorption spectra of those stratified devices before Al back electrode deposition.

In conclusion, we demonstrate that TiO-RGO and ZnO-RGO can be used as efficient ETLs to achieve device performances above that of other commonly used ETL materials.^4^,^19^,^20^ Based on the analysis of the data acquired from electron only devices, the hybrid metal oxide loaded with graphene ETLs demonstrate enhanced electrical properties. High performance OPV devices were achieved with both graphene loaded versions of metal oxides as ETL but the largest increase was found when using ZnO-RGO. The slightly higher efficiencies observed with the solution processed ZnO-RGO ETL indicates that the optimum device structure for these OPVs is ITO/PEDOT:PSS/PCDTBT:PC_70_BM/ZnO-RGO/Al. These results demonstrate that OPV technology is a step closer towards being an all solution processed technology based on low cost readily available materials. Furthermore, this progress on hybrid graphene-metal oxide materials indicates the potential to fabricate matching bespoke tailored layers for tandem polymer solar cells, thereby paving the way for higher efficiencies and better device life times.

## Experimental Section

*Material Synthesis*: For the Graphene Oxide (GO) synthesis a modified Hummers method^25^–^27^ was used. H_2_SO_4_ (23ml, Fisher Scientific, batch no. 0944541, >95% concentration) was added to a round bottle flask immersed in a dry ice bath to bring the temperate down to 0 ºC. Graphite flakes (1 g, Fisher scientific, batch no. 0438775) were added to the sulfuric acid. After stirring for 10 min at 250 rpm, NaNO_3_ (0.5 g) and KMnO_4_ (3 g) were slowly added separately to the solution to keep the temperature below 20 ºC. The stirring was maintained for 30 min then deionized (DI) water (46 ml) was slowly added, raising the temperature to 90 ºC due to the exothermic nature of the reaction. After 15 min, cold water (70 ml) was added, followed by H_2_O_2_ (10 ml, 30% w/w) and kept stirring for 20 min to stop the reaction. The precipitant was mixed with HCl (100 ml, 5% v/v) and centrifuged for 10 min at 6000 rpm; repeated 5 times for neutralization. Finally, the powder was allowed to dry overnight in a vacuum oven at 50 ºC and 700 mbar.

For the TiO_2_-RGO synthesis, GO (20 mg) was dispersed in deionized water (20 ml) and ultrasonicated for 30 min with a probe sonicator at 35% power. Thereafter, P25 TiO_2_ (200 mg, Degussa batch 4166031598) was added to the solution and stirred for 30 min to ensure a good dispersion. The mixture was loaded into an autoclave vessel and heated in an oven at 180 ºC for 6 h to reduce the GO and bind it to the TiO_2_ (Figure 1a, b). Thereafter, the solution was centrifuged at 6000 rpm for 10 min and the precipitant dried overnight in a vacuum oven at 40 ºC. Similarly, for the preparation of ZnO-RGO (Figure 1c, d) the same synthesis procedure was used by replacing TiO_2_ with ZnO (Fisher Scientific batch Z/1300/S3).

*Device Fabrication*: Organic photovoltaic devices were fabricated based on PCDTBT/PC_70_BM by utilizing metal oxides or the hybrid metal oxides-reduced graphene oxide materials as electron transport layers. The devices were fabricated on pre-patterned ITO glass substrates (sheet resistance 15 Ω □^−1^), cleaned beforehand using ultra sonication in acetone and methanol for 10 min, followed by oxygen plasma (15 SCCM, 5 min, 100 W; Emitect K1050X). A hole transport layer of PEDOT:PSS (Baytron P VP AI 4083) was filtered (0.45μm) and spin coated on top of the ITO to form a 35 nm thick layer after baking at 150 ºC for 10 min. The active layer of PCDTBT:PC_70_BM (1:4) in 1,2-dichlorobenzene:chlorobenzene (3:1) was spin coated on top of PEDOT:PSS at 5500 rpm to form an 80nm layer which was dried at 80 ºC for 10 minutes. The electron transport layer (TiO_2_, ZnO, TiO_2_-RGO and ZnO-RGO) solutions were diluted in anhydrous methanol (1:200) (v/v) and spin coated on top of the active layer, followed by solvent annealing at 80 ºC for 1 min. Finally a 100 nm thick back electrode of aluminum was deposited using thermal evaporation under a pressure of 1.0 × 10^−6^ Torr.

*Electron Only Device Measurements*: To study the drift of electrons perpendicular to the ETL's plane the electron only device method^28^ for n-type metal oxides was used. The device configuration employed for the measurements was SiO_2_/PEDOT:PSS/Al/ETL/Al. The current -voltage scans were taken in the range -0.5 to 0.5 V (see Supporting Information)

*Characterization*: The device performances were measured under Air Mass 1.5 Global (AM1.5G) using a Newport solar simulator calibrated at 1000 W m^−2^. The current density-voltage characteristics and power conversion efficiency (PCE) were measured using a PC controlled Keithley 2425 source meter. The external quantum efficiency (EQE) of the devices was measured using a Bentham PVE300 solar cell characterization system. Raman spectra were acquired using a Renishaw micro-Raman 2000 system with a 782 nm laser at 4 mW. A (50x) optical lens was used to focus the laser spot size down to an approximately 1 μm. The detector integration time was set to 10 seconds and 4 accumulations on the same area were acquired to improve the signal-to-noise ratio. Scanning Electron Microscopy (SEM) imaging of the MO nanoparticles (NPs) was performed in an FEI Quanta 200 environmental, while high resolution cross-section images of the samples was performed in a Hitachi HD2300 transmission electron microscope (TEM) operating at 200 kV. Fourier transform infrared spectroscopy (FTIR) measurements were performed with a Varian 660-IR spectrometer. The X-ray photoelectron spectroscopy (XPS) was performed with an Omicron UHV system equipped with an EA125 hemispherical photoelectron energy analyzer and a XL32 dual anode X-ray source. The samples were loaded in an ultra-high vacuum system (1 × 10^−8^ mbar) and measurements carried out at 15kV and room temperature.
