# Supplementary material for: Hybrid Graphene-Metal Oxide Solution Processed Electron Transport Layers for Large Area High-Performance Organic Photovoltaics
Source: Adv Mater. 2014 Jan 2;26(13):2078–83. doi: 10.1002/adma.201304780 (PMC4286003; doi:10.1002/adma.201304780)
Supplement: Supplementary file 1 — Supplementary [file adma0026-2078-SD1.pdf]

Copyright WILEY-VCH Verlag GmbH & Co. KGaA, 69469 Weinheim, Germany, 2013.

ADVANCED MATERIALS

Supporting Information

for *Adv. Mater.*, DOI: 10.1002/adma.201304780

Hybrid Graphene-Metal Oxide Solution Processed Electron Transport Layers for Large Area High-Performance Organic Photovoltaics

*Michail J. Beliatis, Keyur K. Gandhi, Lynn J. Rozanski, Rhys Rhodes, Liam McCafferty, Mohammad R. Alenezi, Abdullah S. Alshammari, Christopher A. Mills, K. D. G. Imalka Jayawardena, Simon J. Henley, and S. Ravi P. Silva**

Supporting Information for manuscript entitled "Hybrid Graphene-Metal Oxide Solution Processed Electron Transport Layers for Large Area High Performance OrganicPhotovoltaics"

Michail J. Beliatis,KeyurK. Gandhi, Lynn J. Rozanski,Rhys Rhodes,Liam McCafferty,Mohammad R. Alenezi ,Abdullah S. Alshammari,Chris Mills, K. D. G. ImalkaJayawardena,Simon J. Henley and S. Ravi P. Silva*

For PCDTBT:PC70BM film the RMS roughness was 0.5nm and max peak height 2.79nm

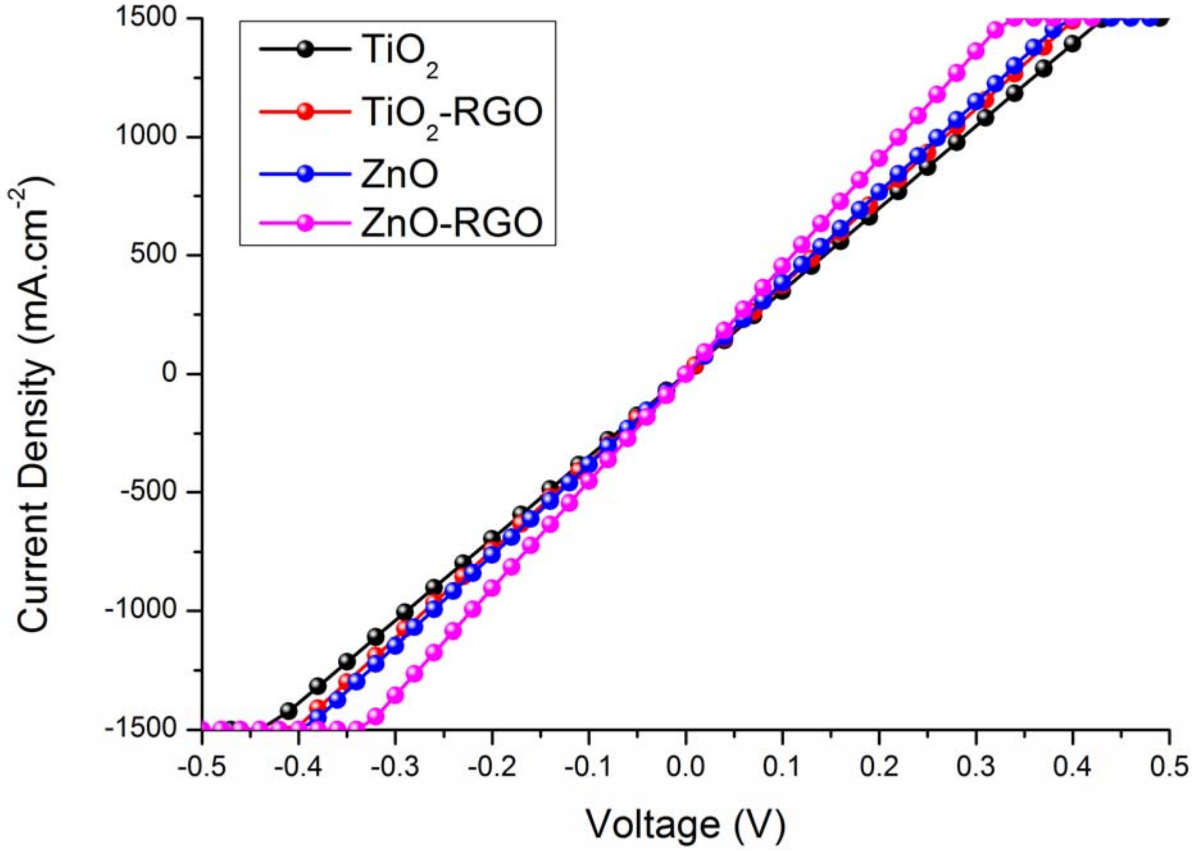

Figure 1. Current density – voltage curves from electron only devices characterize to determine the conductivity perpendicular to the electron transport layer (ETL) plane using TiO_2, ZnO, TiO_2/RGO, ZnO/RGO for ETL.

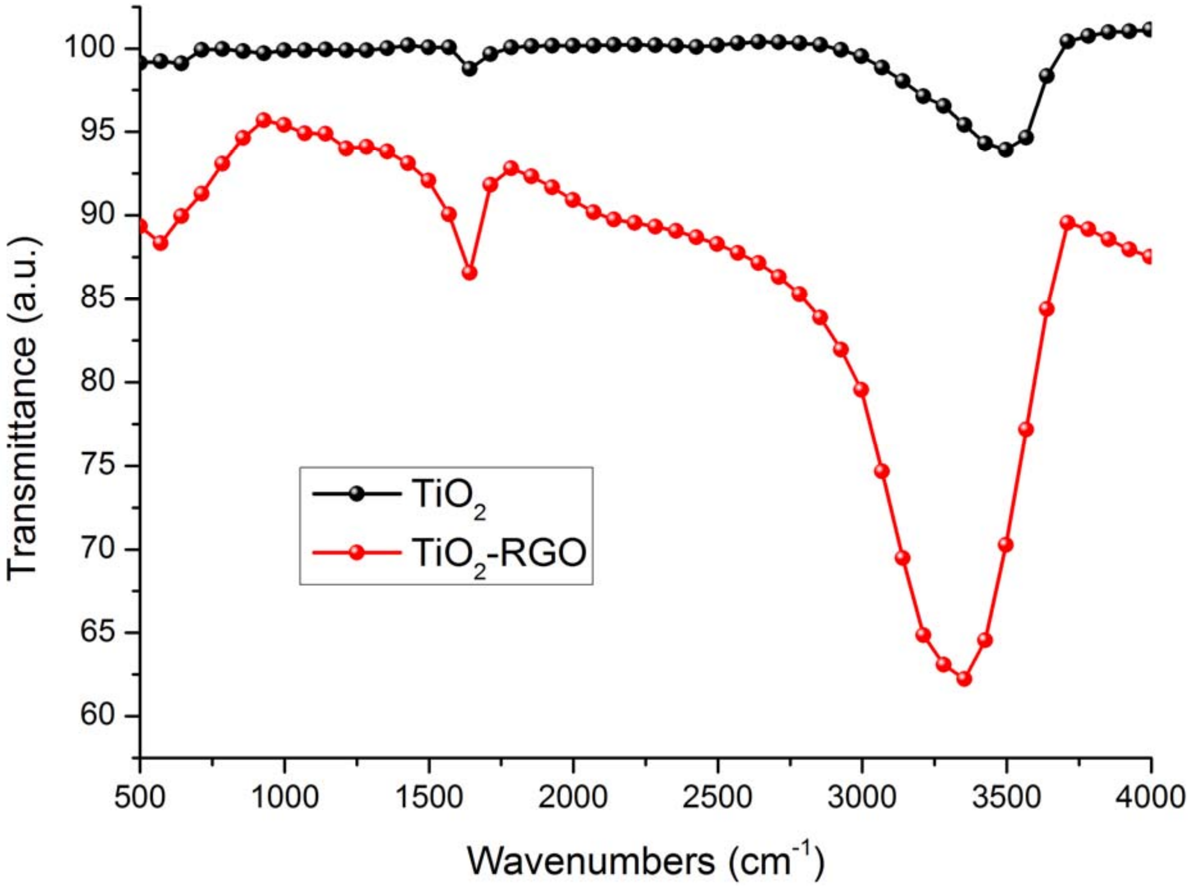

Figure 2.FTIR spectra of TiO_2(black line) and TiO_2 loaded with reduced graphene oxide (red line). Pristine TiO_2 contains peaks at ~3700-3000 cm^{-1}, 1640 cm^{-1}, and a smaller, broad peak from 700-500 cm^{-1}. The peak at 3450 cm^{-1} appears to possess a small shoulder at ~3250 cm^{-1}, attributed to the presence of water (most likely absorbed from the atmosphere[1]) and surface hydroxyl groups[2] respectively. The presence of water within the system is also indicated by the weak peak at ~1640 cm^{-1}, which can be assigned to the water bending vibration[3].The small, broad peak from 700-500 cm^{-1} has been attributed to a Ti-O-Ti stretching vibration[4]. XPS investigations carried out previously[5] have shown the disappearance of carboxylate groups within the sample coupled with the formation of O=C-O groups, which are believed to bond the TiO_2 and RGO. The peak at 1650 cm^{-1} can be attributed to the stretching vibrations of C=C groups, present in the graphene skeleton, while the peak at ~3400 cm^{-1} can again be attributed to O-H groups present as in carboxylate and hydroxyl groups on the surface of the RGO[2, 6].

Table 1. Average PCE values for single solar ($28 mm^2$) calculated using 5 different devices per ETL.

**Electron Transport Layer (ETL)**	**Average PCE%**	**Std. dev.**
TiO_2	6.10	0.29
TiO_2-RGO	6.4	0.18
ZnO	6.18	0.07
ZnO-RGO	6.63	0.09

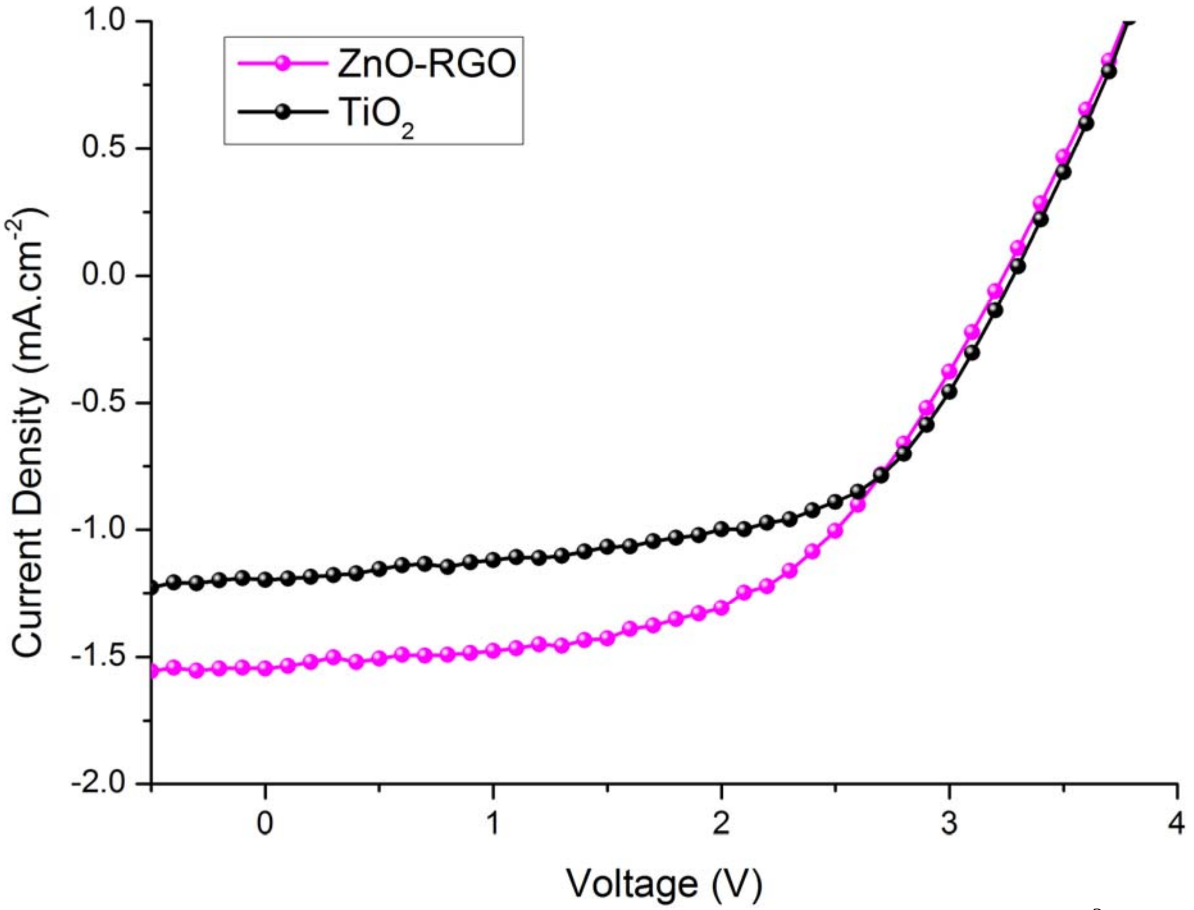

Figure 3. Current density-voltage curve for the module cells(active area 3920 mm^2) tested under 1 sun, for ZnO-RGO ETL (pink line) V_{oc} of 3.23V, J_{sc} 1.54 mA cm^{-2}, FF 54.1%, and PCE2.69% and for TiO_2 ETL (black line) V_{oc} of 3.27V, J_{sc} 1.2 mA cm^{-2}, FF 57%, and PCE 2.23%

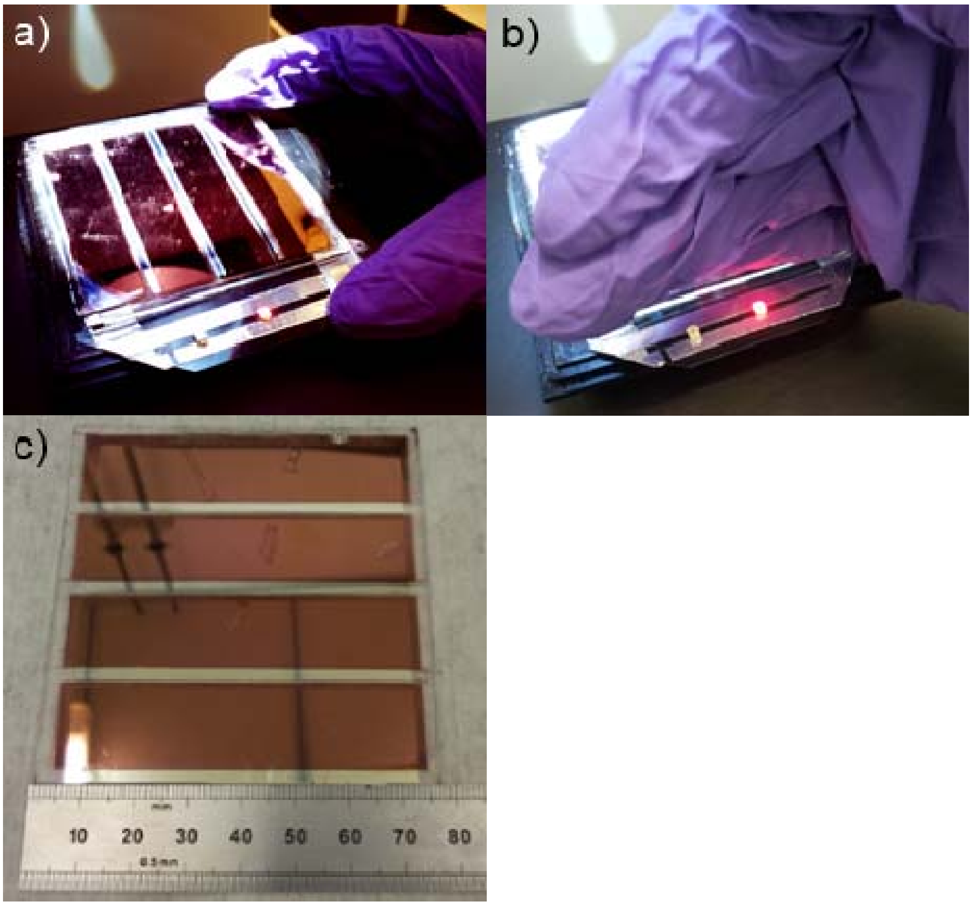

Figure 4.a, b) Module device consisting of four single cells interconnected in series to provide adequate power for powering a commercial LED on plastic substrate, c) real dimensions of the module cell measured with a ruler for reference.

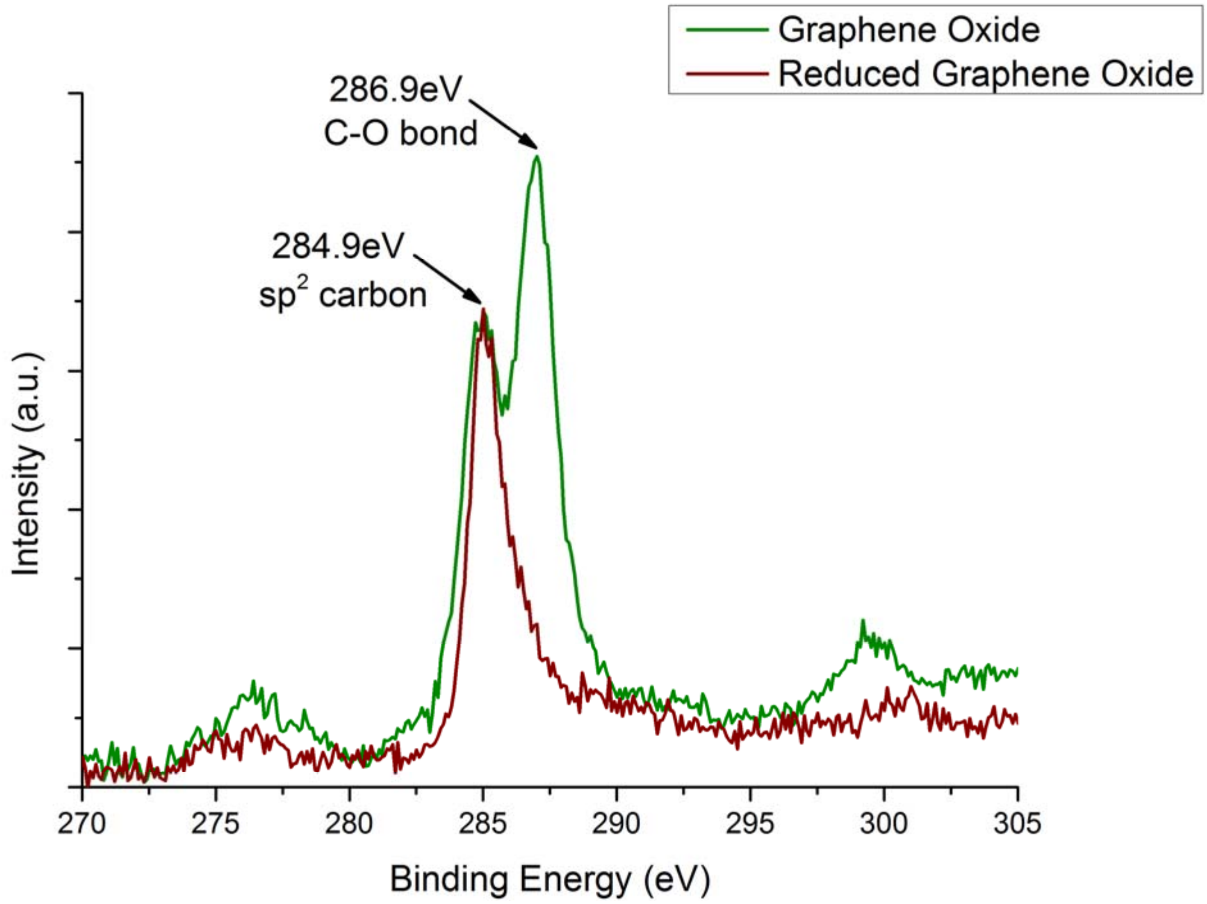

Figure 5. XPS spectra ofgrapheneoxide (green line) and reduced grapheneoxide (red line) showing the different oxidation levels of thegraphene oxide before and after reduction. The peak at 284.9eV denotes the sp^2 carbon bond characteristic of the graphitic material in the sample and appearsin both samples. The peak at 286.9eV is attributed to single bonded carbon-oxygen (C-O) group and appears only in thegraphene oxide sample prior to reduction.

[1] V. Baglio, R. Ornelas, F. Matteucci, F. Martina, G. Ciccarella, I. Zama, L. G. Arriaga, V. Antonucci, A. S. Aricò, Fuel Cells 2009, 9, 247.
[2] G. Williams, B. Seger, P. V. Kamat, ACS Nano 2008, 2, 1487.
[3] S. E. Lappi, B. Smith, S. Franzen, Spectrochimica Acta Part A: Molecular and Biomolecular Spectroscopy 2004, 60, 2611.
[4] H. Zhang, X. Lv, Y. Li, Y. Wang, J. Li, ACS Nano 2009, 4, 380.
[5] Q. Xiang, J. Yu, M. Jaroniec, Nanoscale 2011, 3, 3670.
[6] D. Li, M. B. Muller, S. Gilje, R. B. Kaner, G. G. Wallace, Nat Nano 2008, 3, 101.
